# Supplementary figures and images for: Development of an inexpensive matrix-assisted laser desorption—time of flight mass spectrometry method for the identification of endophytes and rhizobacteria cultured from the microbiome associated with maize
Source: PeerJ. 2021 May 28;9:e11359. doi: 10.7717/peerj.11359 (PMC8166240; doi:10.7717/peerj.11359)

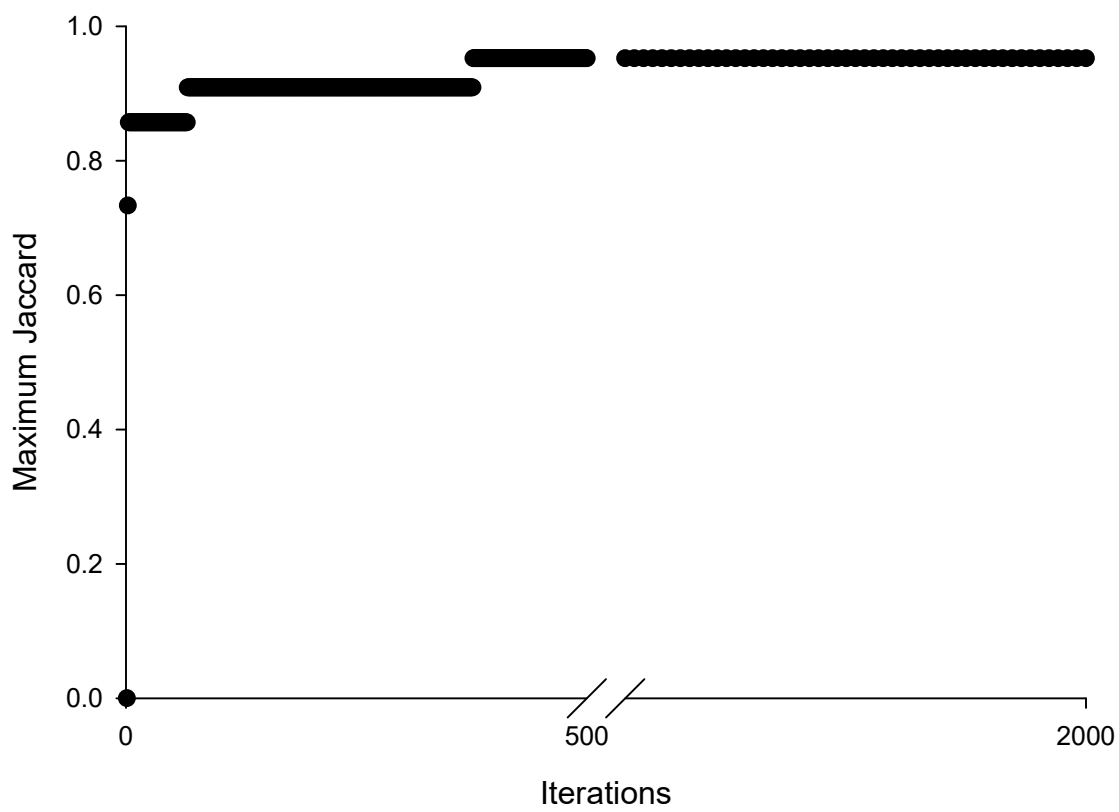

Supplement: Supplemental Information 7 — Y-axis presents number of peaks shared. Points correspond to values calculated with different iterations of randomly selected parameters used to align spectra (see Methods). [file peerj-09-11359-s007.pdf]

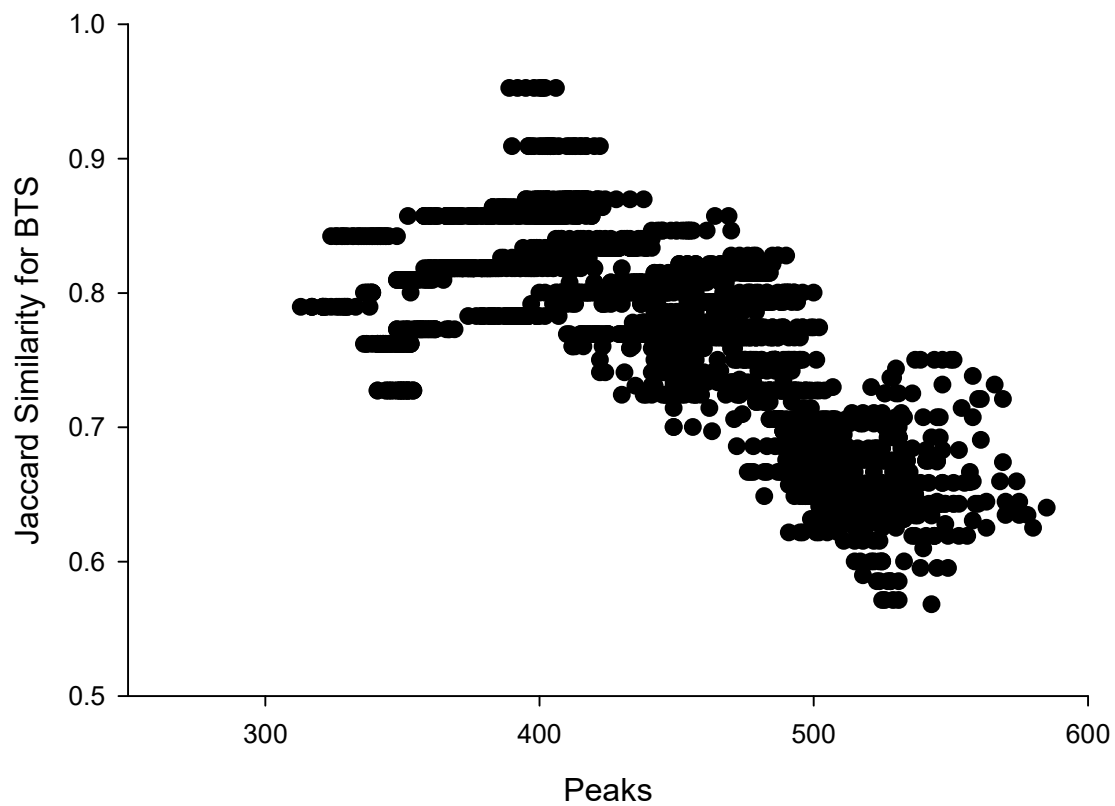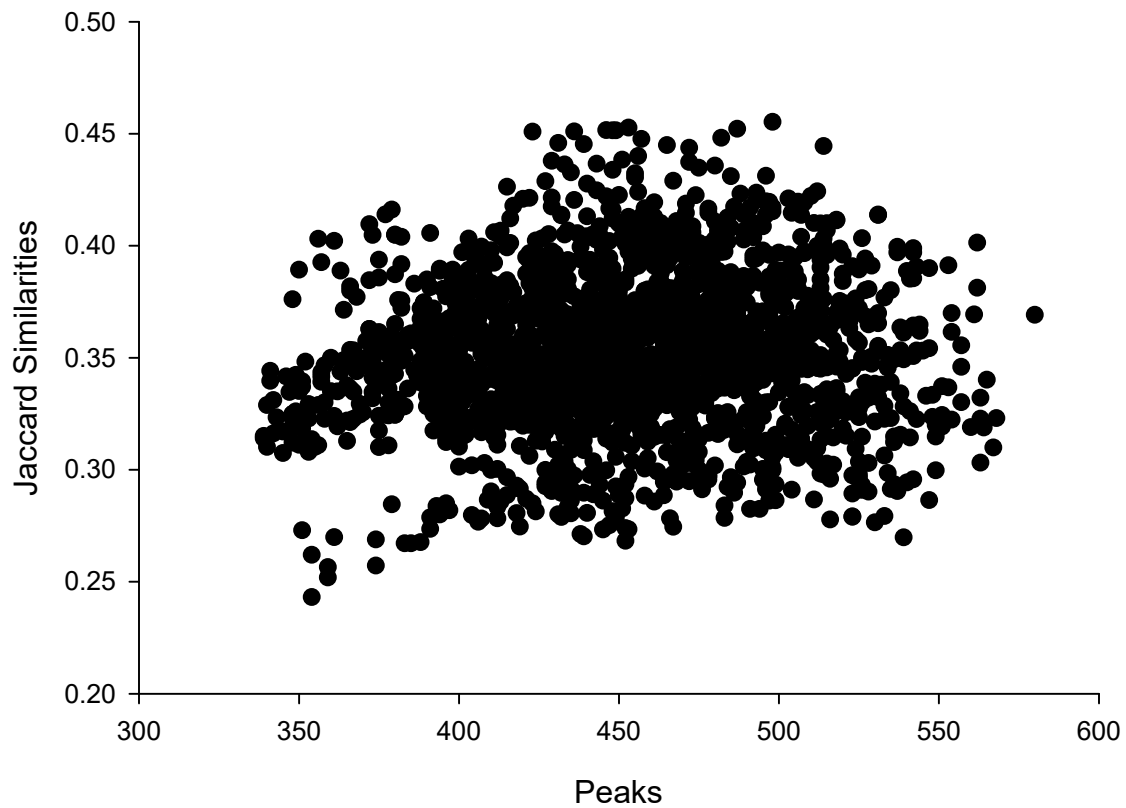

Supplement: Supplemental Information 8 — Y-axis presents number of peaks shared. X-axis presents peaks. Points correspond to values calculated with different iterations of randomly selected parameters used to align spectra (see Methods). This figure shows the similarity between mass spectra generated from replicate spots of the bacterial test standard (top) and for pairwise comparisons of mass spectra generated from isolates with highly similar 16S rRNA gene sequences (bottom). [file peerj-09-11359-s008.pdf]

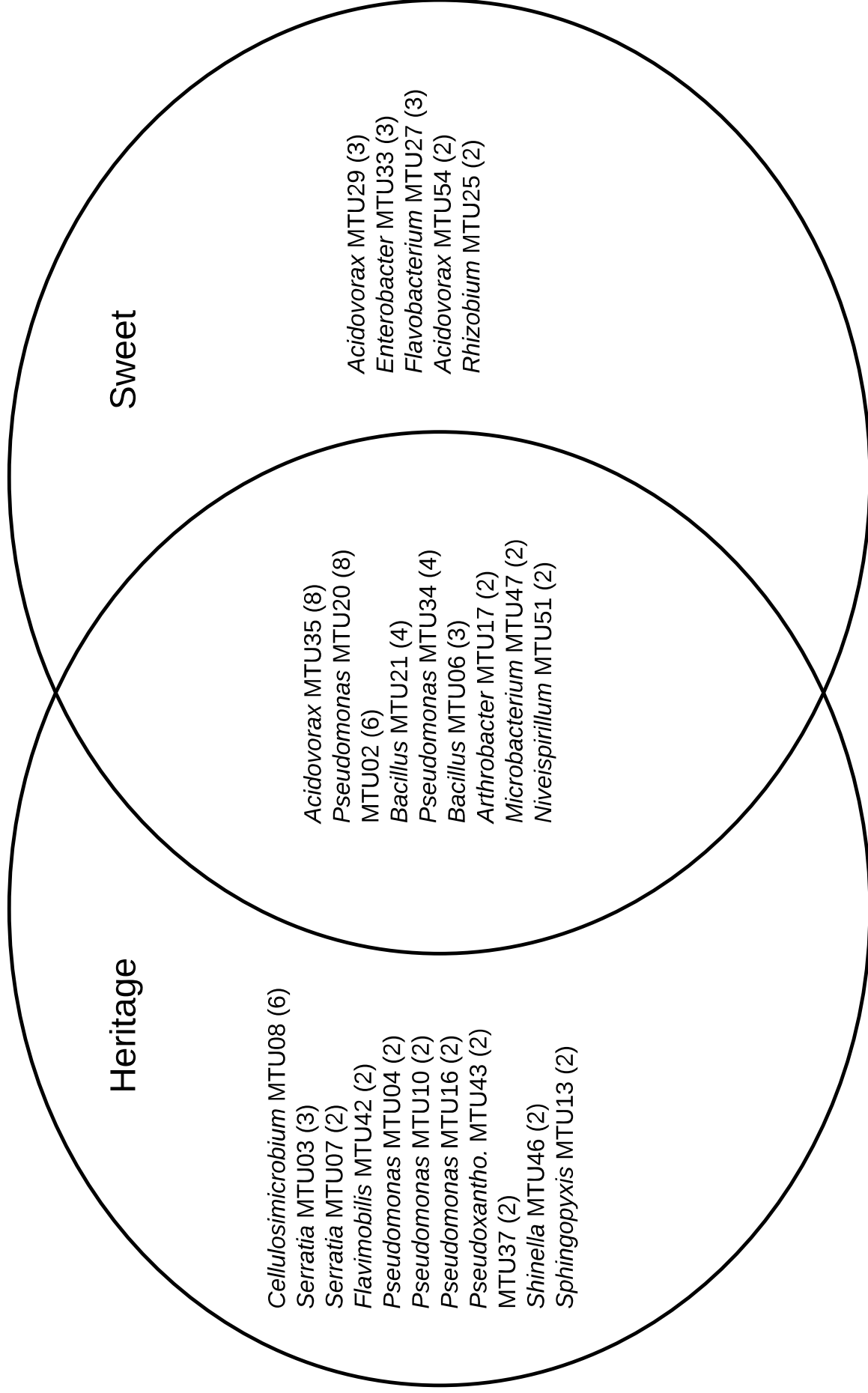

Supplement: Supplemental Information 9 — The number of isolates clustered in each MTU is indicated. Singletons are not shown. [file peerj-09-11359-s009.pdf]
